# The Impact of Composition and Morphology on Ionic Conductivity of Silk/Cellulose Bio-Composites Fabricated from Ionic Liquid and Varying Percentages of Coagulation Agents

**DOI:** 10.3390/ijms21134695

**Published:** 2020-06-30

**Authors:** Bailey Blessing, Cory Trout, Abneris Morales, Karleena Rybacki, Stacy A. Love, Guillaume Lamoureux, Sean M. O’Malley, Xiao Hu, David Salas-de la Cruz

**Affiliations:** 1 Department of Chemistry, Rutgers University, Camden, NJ 08102, USA; bvb9@camden.rutgers.edu (B.B.); am2033@camden.rutgers.edu (A.M.); guillaume.lamoureux@rutgers.edu (G.L.); 2 Department of Physics, Rutgers University, Camden, NJ 08102, USA; cjt122@scarletmail.rutgers.edu (C.T.); omallese@camden.rutgers.edu (S.M.O.); 3 Center for Computational and Integrative Biology, Rutgers University, Camden, NJ 08102, USA; kcr60@scarletmail.rutgers.edu (K.R.); sw524@scarletmail.rutgers.edu (S.A.L.); 4 Department of Physics and Astronomy, Department of Biomedical Engineering, Rowan University, Glassboro, NJ 08028, USA; hu@rowan.edu

**Keywords:** cellulose, silk, morphology, ionic conductivity, X-ray scattering, β-sheets, crystallinity

## Abstract

Blended biocomposites created from the electrostatic and hydrophobic interactions between polysaccharides and structural proteins exhibit useful and unique properties. However, engineering these biopolymers into applicable forms is challenging due to the coupling of the material’s physicochemical properties to its morphology, and the undertaking that comes with controlling this. In this particular study, numerous properties of the *Bombyx mori* silk and microcrystalline cellulose biocomposites blended using ionic liquid and regenerated with various coagulation agents were investigated. Specifically, the relationship between the composition of polysaccharide-protein bio-electrolyte membranes and the resulting morphology and ionic conductivity is explored using numerous characterization techniques, including scanning electron microscopy (SEM), Fourier transform infrared spectroscopy (FTIR), thermal gravimetric analysis (TGA), differential scanning calorimetry (DSC), X-ray scattering, atomic force microscopy (AFM) based nanoindentation, and dielectric relaxation spectroscopy (DRS). The results revealed that when silk is the dominating component in the biocomposite, the ionic conductivity is higher, which also correlates with higher β-sheet content. However, when cellulose becomes the dominating component in the biocomposite, this relationship is not observed; instead, cellulose semicrystallinity and mechanical properties dominate the ionic conduction.

## 1. Introduction

Polysaccharides and proteins, when blended together, form biocomposites that can lead to new and useful properties and technologies such as scaffolds, drug delivery capsules, and bio-electrolyte membranes [1,2]. These materials could ideally be used in the human body as they have high biocompatibility and hold promise in the development of medical batteries. By furthering the understanding of the relationship between the ionic conductivity and the morphology, it would be possible to tune these biocomposites to a variety of applications. 

Cellulose is a structural polymer and the most abundant polysaccharide on earth [3]. The natural polymer is formed of several repeating glucose residues connected through β-(1–4) glycosidic linkers [4]. The microcrystalline natural form, with a parallel arrangement of strands, is called cellulose I; several other versions of cellulose can exist as well, such as cellulose II and III [5]. Cellulose II contains antiparallel packing [6], and is often processed from cellulose I through regeneration or the use of caustic chemicals [7]. Such techniques are also used to create cellulose III [5]; however, the process is complicated since cellulose is resistant to breaking apart due to its complex hydrogen-bonding network [7]. Specifically, intra- and intermolecular hydrogen bonds occur within this structure due to the numerous hydroxyl groups, which make the polysaccharide resistant to breakage [7,8].

Cellulose can be blended with *Bombyx mori* silk, a spin fiber coated in sericin proteins. This sericin coating is removed with different chemicals to ensure only the pure fibroin remains. These fibroins contain the amino acids glycine, alanine, and serine in the repeating formation [GAGAGS]_n_ [9]. Some of the notable characteristics of this protein include high biocompatibility, slow degradability, toughness, and high tensile strength [9,10]. Toughness and high tensile strength may result from the crystalline regions within the fibroin, which experience strong attraction through the hydrogen bonding of the C-O and N-H groups on the amino acids, which sometimes arrange in an antiparallel direction [11,12]. Within the protein, there are secondary structures, including alpha helices, random coils, side chains, and turns. However, one of the most essential secondary structures that can lead to changes in the physicochemical properties is β-sheets. Interestingly, random coils and alpha helices can be transformed into β-sheets using alcohol-based solutions [9,10,13,14]. 

Both of these materials, cellulose and silk, are considered to fall under the category of biomaterials, which are defined as materials that interact with biological systems and can be both manufactured and natural [1]. Being able to tune the thermal, conductive, and morphological properties from their native state into various biomaterial-based applications is imperative but presents challenges in terms of dissolution and fabrication. One way to dissolve these natural macromolecules, without affecting their molecular weight, is through the use of various ionic liquids [15,16,17,18]. These are molten salts at room temperature with high ionic conductivity, wide electrochemical windows, and good thermal stability [19,20]. During the dissolution process, the hydrogen bonding is disturbed by associating the anion and cation of the ionic liquid with the hydrogen and oxygen of the hydroxyl groups of cellulose [19,21]. Once the silk chains undergo a similar process, these two biopolymers will interact through different types of interactions in solution: hydrophobic-hydrophobic, electrostatic, and hydrogen bonding [16,22]. A coagulation agent, such as ethanol or water, is then used to remove the ionic liquid by causing the anions of the salt to move into the liquid and out of the biocomposite. Typically, only a small amount of residual ionic liquid is left. Once the ionic liquid is removed, the coagulant now creates a phase separation of liquid and hydrogel regions. Upon drying, the coagulant is removed, and as a result, the silk and cellulose can aggregate in its absence [15,16,17,23].

Ionic conductivity in solid polymer electrolytes is dependent on the material’s physicochemical properties, including morphology, which can directly affect ion diffusion and dissociation processes [24]. For example, natural silk was found to be considerably more conductive than natural cellulose, which demonstrates the significance that composition may play in connection with conductivity [25]. Additionally, a previous study demonstrated the effects of two ionic liquids, 1-Ethyl-3-methylimidazolium acetate (EMIMAc) and 1-Ethyl-3-methylimidazolium chloride (EMIMCl), and two coagulation agents, 25% ethanol and 25% hydrogen peroxide, on resulting morphological, thermal, mechanical, and ionic conductivity properties [18]. The study revealed that the ionic conductivity was dependent on β-sheet content. Higher β-sheet content corresponded to higher ionic conductivity, a conclusion observed by others as well when using different materials and processing conditions [18,26]. 

Based on these studies, a general hypothesis can be stated that the conductivity would correlate with the dominating polymer, such that higher silk content would lead to higher conductivity, and higher cellulose content would lead to lower conductivity. Therefore, in this study, the effects of varying composition on conductivity as well as other morphological, thermal, and mechanical properties will be investigated. Two different ratios of silk and cellulose biocomposites used include 25% silk/75% cellulose and 75% silk/25% cellulose. Pure silk and pure cellulose samples were also created for comparison purposes. All samples, including silk, cellulose, and the biocomposites, were dissolved in 1-ethyl-3-methylimidazolium acetate and coagulated in various agents. It is evident from the data that the composition, as well as the coagulation agent, produce morphological, thermal, mechanical, and conductive variations.

## 2. Results and Discussion

Morphological, thermal, mechanical, and ionic conductivity differences are demonstrated in the qualitative and quantitative data obtained by varying the coagulation agents as well as biopolymer ratios. Films regenerated with a higher percentage of cellulose were less flexible than those with more silk. Morphological differences correlated with changes in secondary structure calculations, mechanical properties, as well as ionic conductivity, and are illustrated in the following characterization tests.

### 2.1. Fourier Transform Infrared Spectroscopy

**Figure 1:**
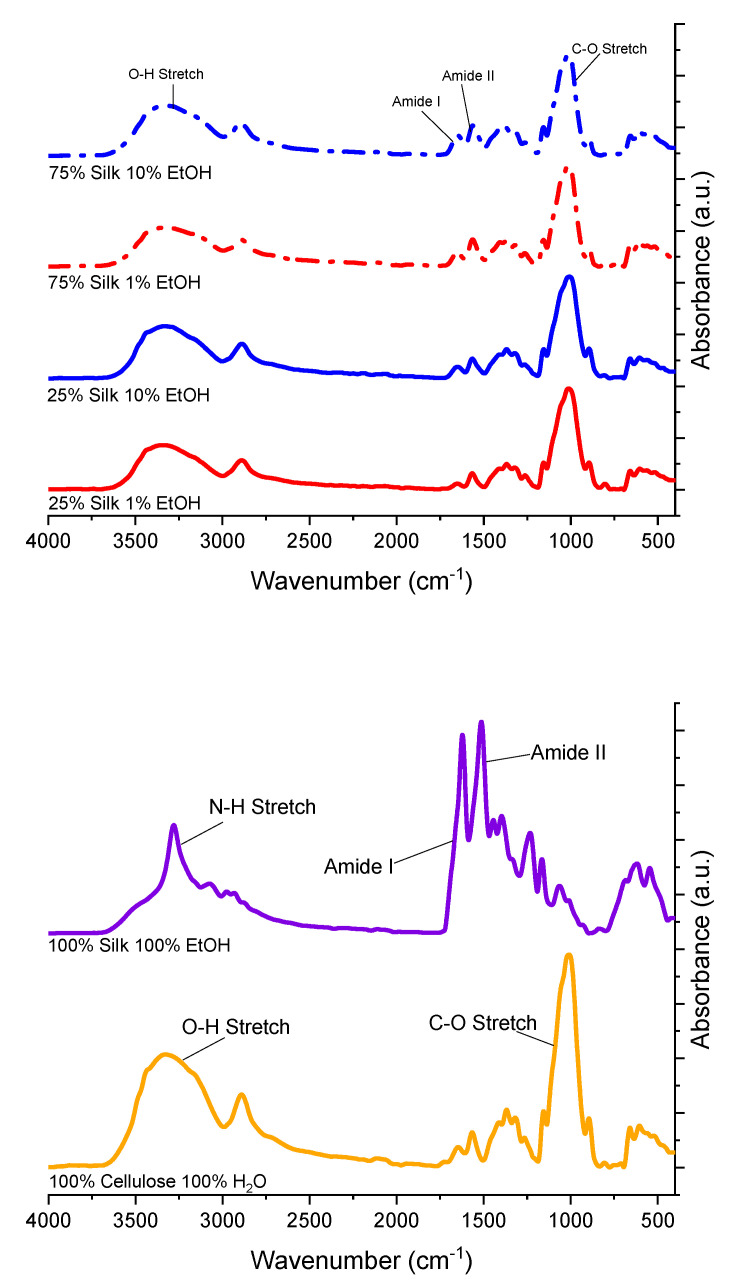
Fourier transform infrared spectroscopy (FTIR) spectra of regenerated silk and cellulose samples, as well as silk/cellulose biocomposites with varying composition ratios and coagulation agents.

Fourier transform infrared spectroscopy (FTIR) was used to identify secondary protein structures within the regenerated biomaterial films, and to ensure proper blending. Figure 1 shows the normalized IR spectra of the six biopolymer films. The spectra were normalized to easily locate the various functional groups within the samples, as well as to compare peak linewidth. In nearly all of the spectra, the two most pronounced peaks can be seen at approximately 1030 cm^−1^ and from 3550 to 3000 cm^−1^, which are stretching modes corresponding to C-O and O-H functional groups, respectively, found in cellulose. All of the spectra corresponding to the films coagulated with 1 and 10% ethanol look very similar in terms of peak breadth as well as the presence of different functional groups. Specifically, these four biocomposites contain the amide I and II regions, labeled in the first spectrum in Figure 1. Furthermore, they all have a small shoulder peak at approximately 1160 cm^−1^, which corresponds to the C-N stretch in an imidazolium ring from the ionic liquid. Since this peak is small and not very pronounced, it is possible there may be only a small amount of residual ionic liquid leftover in the system after using the coagulation agent. 

Compared to the other four spectra, there are noticeable differences seen in the 100% regenerated cellulose and silk spectra. The amide regions are very pronounced in the 100% regenerated silk film, and the N-H stretch peak is also very pronounced. This is seen in the FTIR spectrum of pure *Bombyx mori* silk fibroin and is related to the amine group [16]. When looking at the 100% regenerated cellulose spectrum compared to those of the four mixed films, it looks very different. Specifically, the C-O peak is much more pronounced than in the other films, and the O-H peak is also more distinct. This spectrum looks more similar to the silk/cellulose biocomposites than the 100% regenerated silk spectrum does.

The amide I region was analyzed from 1705 cm^−1^ to 1595 cm^−1^ using Fourier Self-Deconvolution [27], and the secondary structure content from this analysis is summarized in Table 1. When looking at all five secondary structure types, it is clear that the side chains vary the least between all samples, specifically by only 5.27%. This is followed by turns that differ by a maximum of 10.48% and then alpha helices, which vary by 11.47%. Random coils had a slightly higher percentage of change, with 23.47% difference between the 100% regenerated silk film and 25% silk film coagulated with 1% ethanol. Finally, β-sheet content differed by the highest percentage, with there being a 32.74% difference between the 100% regenerated silk film and 25% silk film coagulated with 1% ethanol. If the silk/cellulose biocomposite films are compared without the 100% regenerated silk film, there are no drastic differences in secondary structures. The only secondary structure that varies more than the others is the random coils due to the 25% silk coagulated with 1% ethanol sample having a higher percentage of approximately 39% compared to the other mixtures. If the 100% silk film is used for comparison purposes, it is interesting to see that in three of the five secondary structures, this sample had the lowest percentage when compared to the mixed films. The sample was only greater in side chains and β-sheets when compared to the other biocomposites. The increase in β-sheet content was expected since this film was not only made of 100% silk but was also coagulated with 100% ethanol, which is shown to increase β-sheet content as a function of percentage [9]. β-sheet content differs between all of the samples listed in Table 1 and is also connected to the ionic conductivity, which is discussed in a later section. When looking at the different ratios, the 75% silk samples have higher β-sheet content compared to the 25% silk samples. This may be a result of these samples having a higher silk content, although the percentages are not drastically higher than the 25% silk samples. When looking at the differences in β-sheet content between the samples of the same composition, the 75% silk films have less of a difference than the 25% silk films. The changes in this particular secondary structure are fascinating when correlating it with ionic conductivity. 

### 2.2. Scanning Electron Microscopy

**Figure 2:**
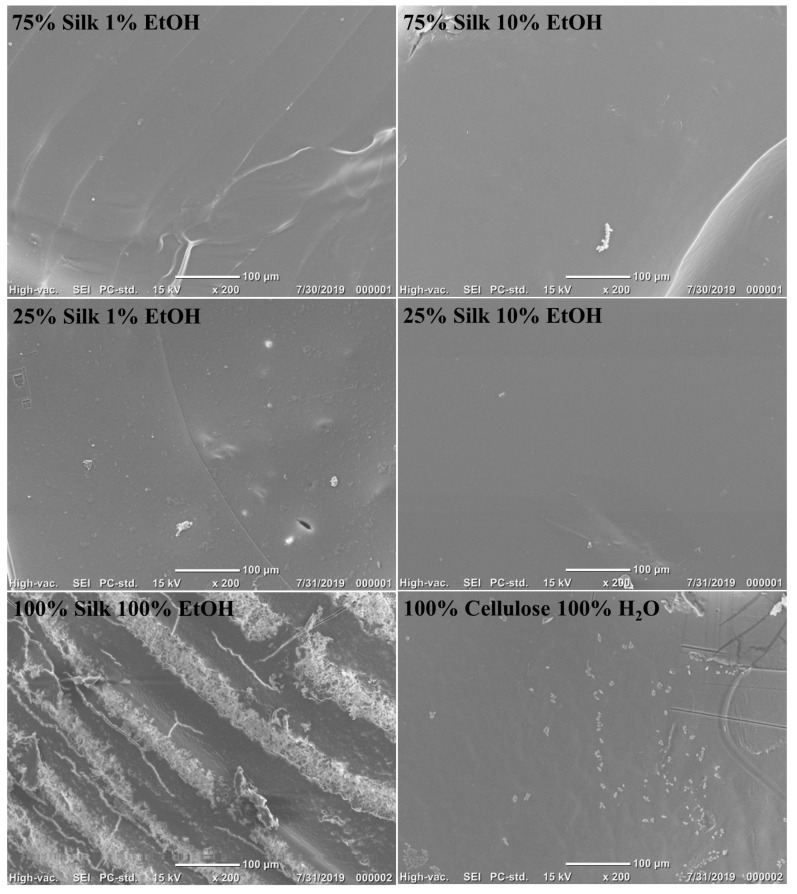
Scanning electron microscopy (SEM) images of 100% regenerated films and varied biopolymer ratio samples using various coagulation agents.

Topographical and morphological properties of the regenerated silk/cellulose biocomposites seen in Figure 2 were studied using Scanning Electron Microscopy (SEM). All films, except for the 100% silk film, generally look very similar to one another at the surface level. Both films coagulated with 10% ethanol look almost entirely smooth on the surface, with the 75% silk film only showing one distinct ridge running along the bottom right corner of the imaged surface. The films coagulated with 1% ethanol look slightly different. The 75% silk film has slight ridges running across the entire surface of the film, while the 25% silk film demonstrates areas with small bumps and a few shallow spheres. In comparison to both films coagulated with 10% ethanol, the films coagulated with 1% ethanol are not as smooth and uniform on the surface. When looking at the films created with only one polymer, the 100% cellulose film looks relatively smooth, but also contains small bumps or pinholes on the surface. Compared to all the other films, the 100% silk film is the most dissimilar. This is apparent in the image presented where striations are along the entire surface of the film, and the silk fibers are visible throughout. It also looks as if there are fine cracks on the surface. The effect of various biopolymer ratios, as well as coagulation agents on the topography of the films, is evident when looking at these images presented in Figure 2.

### 2.3. Thermogravimetric Analysis

**Figure 3:**
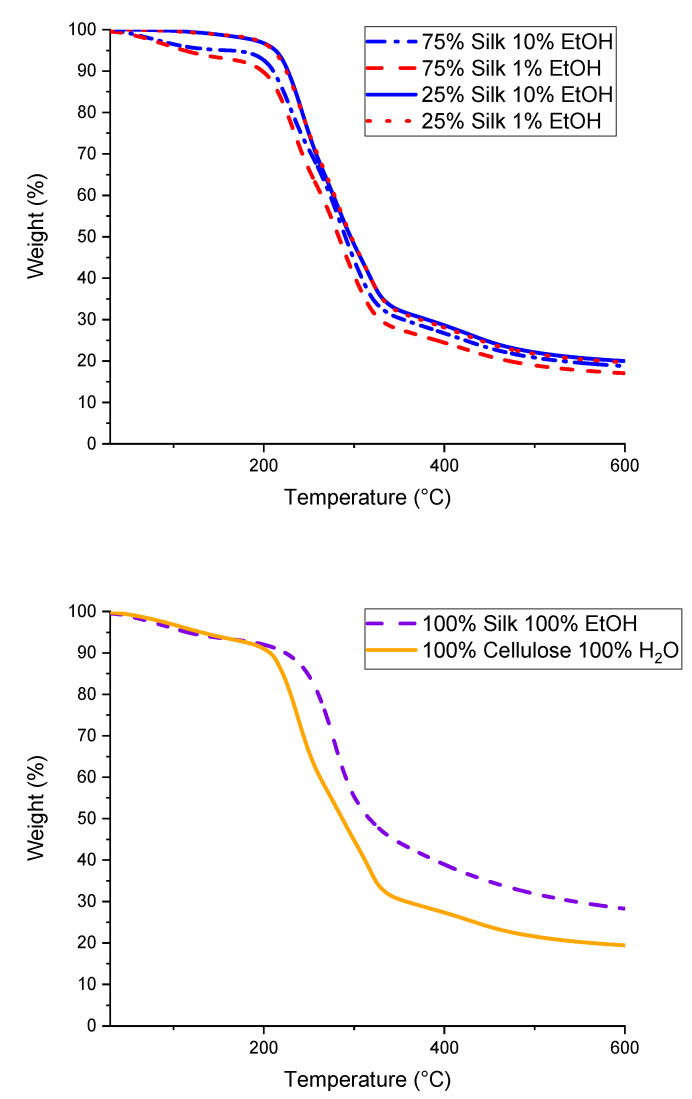
Thermograms of silk/cellulose biocomposites with varied compositions and 100% regenerated samples.

**Figure 4:**
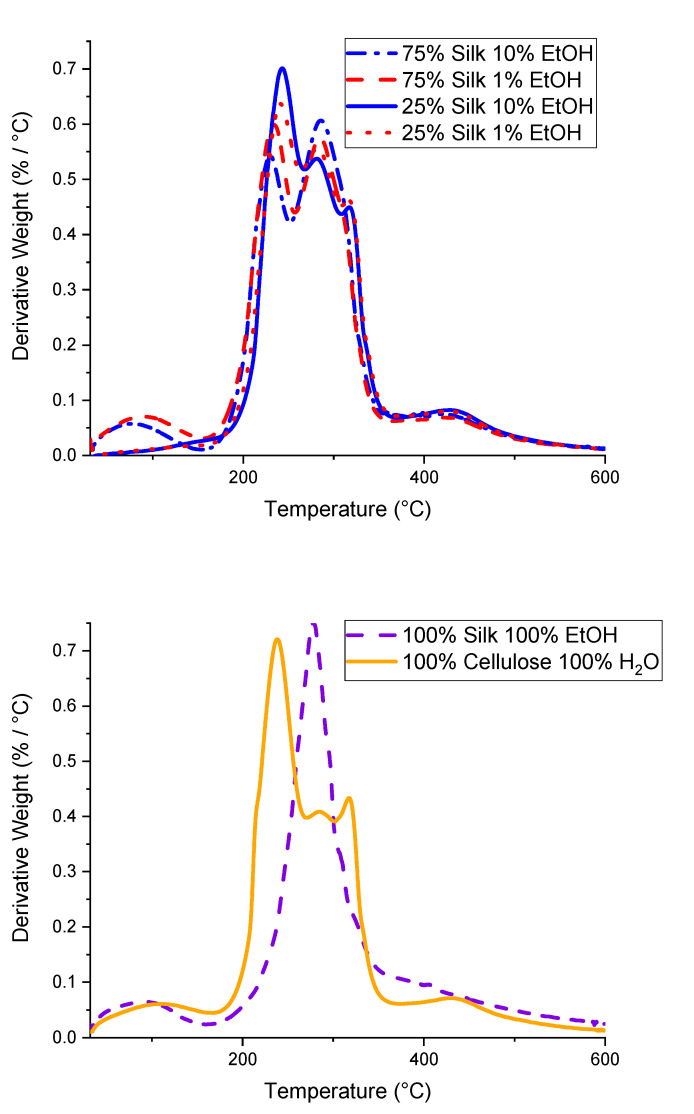
Derivative weight-loss percentage plots of the varied composition biocomposite films as well as 100% regenerated samples, used to determine *T*_∆Max_ as well as other characteristic temperatures.

The thermograms obtained from thermal gravimetric analysis (TGA) analysis of six different biopolymer films are displayed in Figure 3. Figure 4 differs in that it demonstrates the derivative weight-loss percentage thermograms, which help determine crucial thermal stability statistics shown in the biocomposites such as the onset and end temperatures, weight-loss percentage, and *T_∆_*_Max_. Utilizing both Figure 3 and Figure 4, the resulting values from these analyses are displayed in Table 2. First, looking at the 100% regenerated silk and cellulose films in Figure 4, it is clear that the 100% silk film demonstrates a single peak thermogram compared to the 100% cellulose film, which shows a trimodal thermogram. As a result, the silk film most likely has fewer interfaces than the cellulose sample [17]. When looking at the onset, end, and *T*_∆Max_ temperatures of these two samples, it is seen that the silk film has an overall higher thermal stability than the cellulose film. The 100% regenerated silk film has a higher onset temperature by 31.6 °C, higher end temperature by 10.4 °C, lower weight percent loss by 11.26%, and only one *T*_∆Max,_ which corresponds to only one peak and therefore has fewer interfaces. In addition, the thermogram for these two regenerated 100% samples show a solvent evaporation peak at 100 °C. This is the result of the regeneration process from their native state. Upon dissolution and coagulation, a hydrogel is formed and its properties will be affected by solubility. When comparing the four mixed biocomposite samples, it is interesting to see a slight pattern in the thermograms in Figure 4. The two films composed of 75% silk, the higher percentage biopolymer in the film, only have two peaks, meaning it is a bimodal thermogram. This differs from the films with 25% silk, the lesser percentage compared to cellulose, which have three peaks, meaning it is a trimodal thermogram. As the silk content is decreased and the cellulose content increased, making it the dominant biopolymer in the system, the thermograms follow the same trend, deviating from the single peak to the three peaks similar to the 100% regenerated cellulose sample, as expected. When studying the onset, end, and weight-loss percentages of these four films, subtle differences are observed. The 75% silk film coagulated with 1% ethanol has the lowest onset temperature compared to the other three samples, specifically 12 °C lower than the highest onset temperature in 75% silk coagulated with 10% ethanol. The 25% silk coagulated with 10% ethanol sample has the lowest end temperature when compared with the other three mixed biocomposites. Additionally, all weight-loss percentages are nearly the same except for the 25% silk film coagulated with 10% ethanol, which is 2.00% less than the highest weight-loss percentage of the mixed samples. One particular observation is the solvent evaporation peak at 100 °C and it is dependent on silk content. Water absorbance is correlated to semicrystallinity. In the X-ray scattering section below, it is demonstrated that the composite with higher cellulose content is more semicrystalline. As a result, we expect that the samples will have lower to no water retention. These results show how the thermal stability of the biocomposites changes as a function of not only composition but also coagulation agents. When the composition is changed, the results of sample analyses tend to fall more in line with the dominant polymer.

### 2.4. Differential Scanning Calorimetry

**Figure 5:**
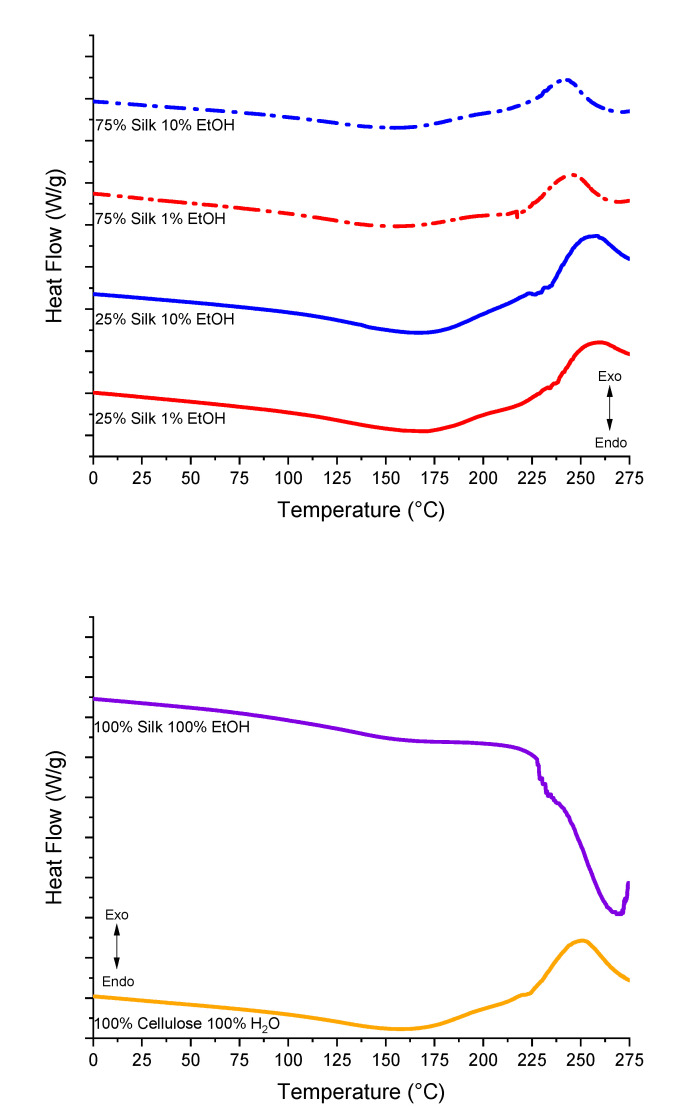
Differential scanning calorimetry (DSC) heat flow scans of the varied composition silk/cellulose biocomposite films and 100% regenerated samples used to determine the glass transition temperatures.

Standard differential scanning calorimetry (DSC) scans for all six samples of either uniform or varying biopolymer percentages are shown in Figure 5. It is interesting to see the crystallization peaks match up relatively closely among the same composition. For example, the crystallization peak for the 75% silk film coagulated with 10% ethanol seems to be around approximately the same temperature as the 75% silk film coagulated with 1% ethanol. This trend holds for the 25% silk samples as well. The 75% silk 10% ethanol sample has this peak at approximately 241 °C compared to 244 °C for the 75% silk 1% ethanol sample. The 25% silk samples both have their peaks at about 256 °C. These peaks are slightly shifted higher in temperature from the 75% silk films. The 100% regenerated cellulose sample has a crystallization peak at approximately 250 °C, but there is no observable crystallization peak for the 100% silk sample. Instead, it contains an endothermic peak at around 269 °C, which could correspond to interface loss in the sample, including its degradation. In addition to observational comparisons, these DSC graphs were analyzed to determine the glass transition temperatures listed in Table 3. The 100% regenerated silk sample has the highest glass transition temperature compared to the other five samples, but not by a drastic amount. All samples seemed to have similar glass transition temperatures. This could be due to the coagulation agents in the different composition samples being only slightly different, enough to produce only subtle effects on the system. The 75% silk samples only differ by 0.52 °C, whereas the 25% silk samples have a more significant difference of 5.77 °C. In a later section, ionic conductivity data is normalized using these glass transition temperatures.

### 2.5. X-Ray Scattering

**Figure 6:**
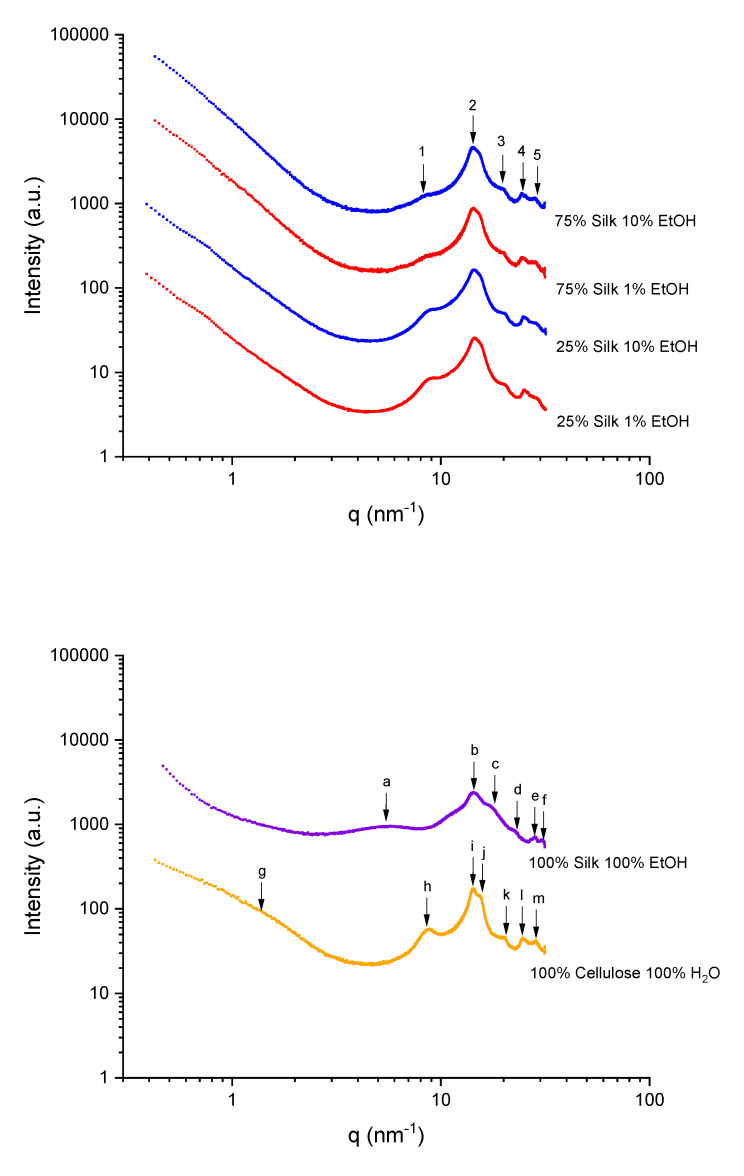
Scattering profiles for varied composition biocomposites and 100% regenerated silk and cellulose samples.

Figure 6 shows the X-ray scattering curves for four biocomposites coagulated in two coagulation agents and regenerated pure samples. The scattering vector and correlation distances for all six samples are recorded in Table 4 and Table 5. The 2D scattering profiles (not shown here) showed isotropic rings for all biocomposites. Table 4 shows each scattering vector with its correlation distance. Qualitatively, the X-ray scattering profiles are very similar for each biocomposite. Only slight changes are observed as a function of increasing silk content. For example, the scattering peak located at *q_1_* = 8.45 nm^−1^ increases to 8.83 nm^−1^ as the silk content increases. Additionally, the broadness of this peak increases. This means that the spacing in between the silk and cellulose carbon chain domains, which is related to the molecular intercalation, increases from 0.71 to 0.75 nm, as calculated by using the *d=2π/q* formula [18]. There is no observable change as a function of the coagulation agent. Interestingly, the higher scattering vector region does not show any nanophase separation. All X-ray scattering profiles show a 45-degree curve. This could mean that the overall system might be separated by distinct interfaces, as similarly reported in the TGA section. The scattering vectors *q_2_*, *q_3_*, *q_4_*, and *q_5_* are related to the cellulose unit cell spacing and the distance between silk β-strands; this region includes the silk I spacings which is a mixture of alpha-helices, β-sheets, and random coils. As expected, as the silk content increases, the sharpness of these peaks also increases.

For 100% regenerated silk in ethanol, the scattering profile shows six scattering peaks. Within the various scattering peaks, the scattering vector, *q_a_* = 5.46 nm^−1^, corresponds to the average of both inter-sheet distances between β-sheets and the size of the β-sheets in the lateral direction. The correlation distance (*d*-spacing) for this peak is calculated by using the *d = 2π/q* formula and was found to be equal to 1.16 nm. The broadness of this peak extended from 2.96 to 8.16 nm^−1^ (0.77 to 2.12 nm). The scattering peaks at 14.45, 17.67, 22.52, 28.66, and 31.40 nm^−1^ correspond to the correlation distances between β-strands and primary structure, especially the silk II crystalline spacings as a result of being modified during dissolution and regeneration [28,29,30,31,32,33,34]. In the 100% regenerated cellulose sample, seven scattering peaks are observed. The first scattering peak is the nanophase separation related to the microfibril located at a scattering vector of *q_g_* = 1.31 (4.80 nm). The cellulose crystallite lateral size is located at *q_h_* = 8.78 (0.72 nm) and the monoclinic unit cell of cellulose I_β_ equatorial lattice planes and its periodicity (14.32, 15.66, 20.56, 24.9, and 29.6 nm^−1^). The correlation distances for these scattering vectors are 0.44, 0.40, 0.30, 0.25, and 0.21 nm [35,36,37,38,39].

### 2.6. Atomic Force Microscopy (Nanoindentation)

**Figure 7:**
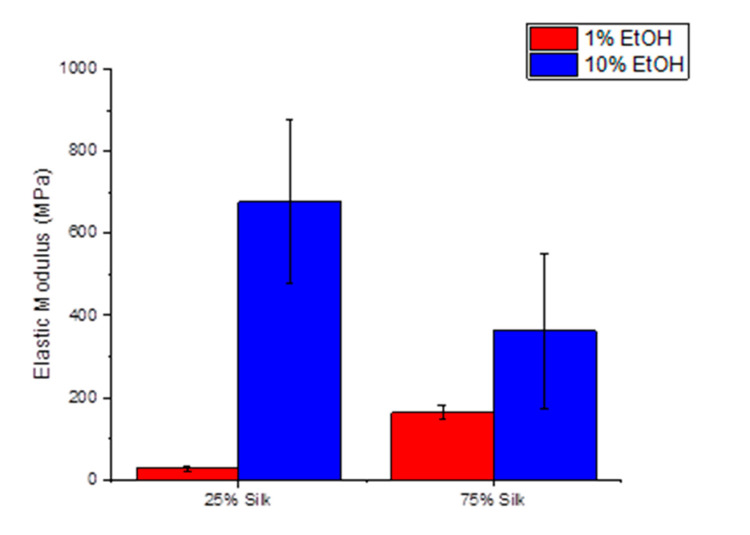
Elastic modulus of the two sets of varied composition silk/cellulose biocomposites coagulated with two different percentages of ethanol.

The elastic modulus of silk/cellulose biocomposites of 25% and 75% silk were measured at 120 °C (near the glass transition temperature) and determined by fitting the load–indentation curves to the Johnson-Kendall-Roberts (JKR) model. In samples of both compositions of silk and cellulose, the elastic modulus increases as the percentage of ethanol is increased from 1% to 10%, as seen in Figure 7. The samples of 25% silk show an increase in the mean elastic modulus from 26 MPa to 676 MPa, while the 75% silk samples show an increase from 163 MPa to 362 MPa. 

It is interesting to note that the coagulation bath, which has the two highest onset temperatures in TGA, also has a higher elastic modulus. These are both the 10% ethanol samples of 25% silk and 75% silk with onset temperatures of 223.4 °C and 225.8 °C, respectively. Additionally, certain determining factors of conductivity relate to these mechanical properties. This includes β-sheet content and semicrystallinity of cellulose, due to cellulose content. First, it is seen that the coagulant with higher β-sheet content in each ratio has a higher elastic modulus. When comparing both 25% silk samples, the 10% ethanol sample has a higher β-sheet content and ultimately has a higher elastic modulus. When looking at both 75% silk samples, the 10% ethanol sample also has a higher β-sheet content, and again a higher elastic modulus. β-sheets are considered to be semicrystalline regions within the silk fiber, so this would make sense; as the β-sheets increase, the elastic modulus does as well, resulting in less flexible and more rigid samples.

### 2.7. Dielectric Relaxation Spectroscopy

**Figure 8:**
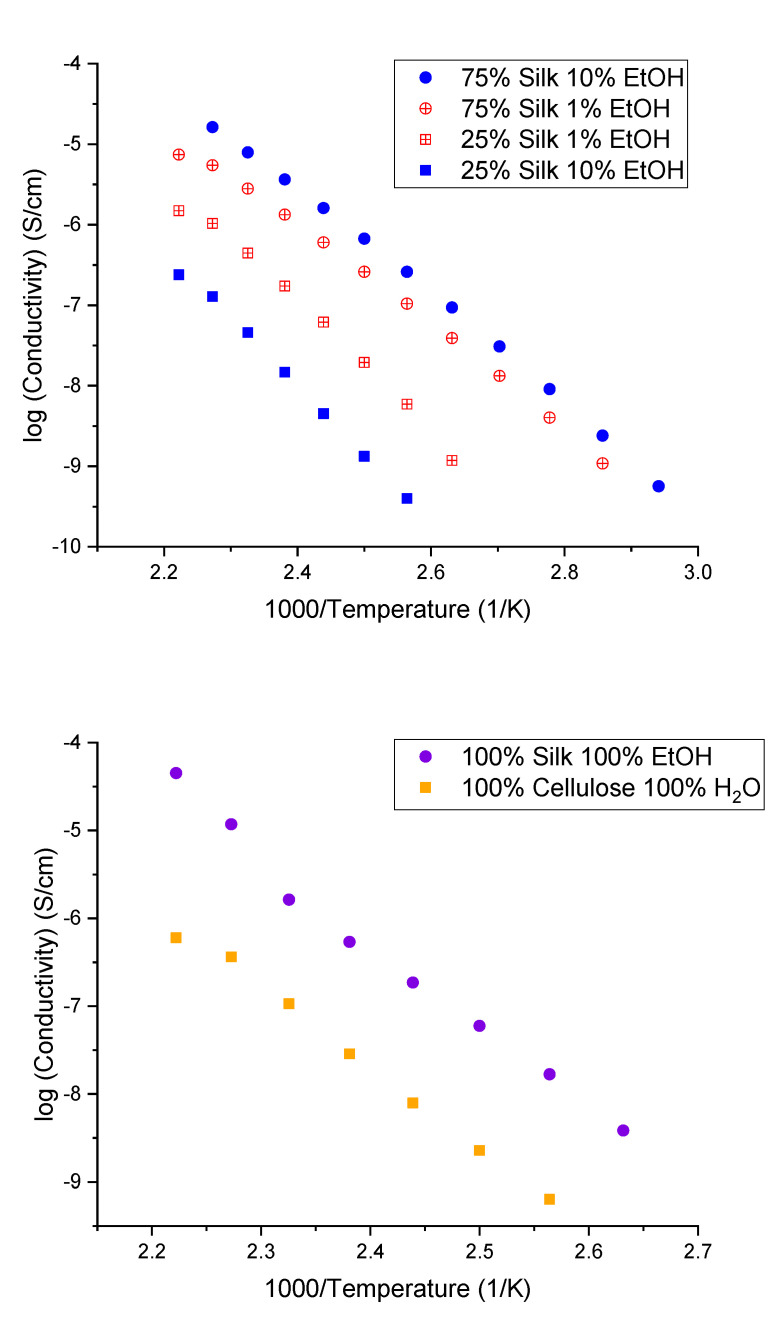
Ionic conductivity versus temperature of the two sets of varied composition silk/cellulose biocomposites as well as 100% regenerated silk, and 100% regenerated cellulose samples.

The ionic conductivities of the six different samples calculated at various temperatures are shown in Figure 8. A negative slope indicates that conductivity increases with temperature. The ionic conductivity was calculated from the measured resistance and physical dimensions of the sample by the following relationship, σ=LAR; where σ is the ionic conductivity, L is the distance between the two inner electrodes, A is the cross-sectional area of the polymer film, and R is the resistance at each temperature calculated using the Nyquist plot. When creating this plot, there will be a semicircle with two *x*-intercepts, one at lower values on the *x*-axis versus higher values. In this case, the *x*-intercept at the high value on the *x*-axis is equal to the resistance [40]. Looking at Figure 8, the ionic conductivity of the 75% silk film coagulated with 10% ethanol is highest, followed by 75% silk coagulated with 1% ethanol, 25% silk coagulated with 1% ethanol, and finally 25% silk coagulated with 10% ethanol. The trend in this data can be explained with not only β-sheet content but also X-ray scattering data and elastic modulus measurements obtained from atomic force microscopy (AFM). First, it was previously discussed that higher β-sheet content corresponded to higher ionic conductivity [18,26]. Although, in general, this may be true, the conductivity is not just a function of β-sheet content but also other morphological properties, and therefore these samples do not strictly obey this rule. The 75% silk film coagulated with 10% ethanol does have the highest β-sheet content with 20.56%, and this is followed by the 75% silk film coagulated with 1% ethanol with 18.24%. The film with the next lowest ionic conductivity based on β-sheet content should be the 25% silk film coagulated with 10% ethanol, which has a β-sheet content of 17.12%. However, this is not the case since this sample has the lowest ionic conductivity. As a result, these observations may be described by the relative silk and cellulose content. In Figure 8 containing the 100% regenerated silk and cellulose samples, it is seen that silk is more conductive than cellulose. As a result, when silk dominates in the mixture, the β-sheet content is the determining factor in ionic conductivity. Additionally, when looking at the 75% silk samples, the sample coagulated with 10% ethanol has a higher conductivity, as well as a higher elastic modulus as compared to 1% ethanol, meaning the sample is more rigid. One would expect this would lead to a decrease in conductivity, possibly due to the decrease in segmental motion within the sample, but the opposite is observed [41]. This may be because the mechanical properties are not as crucial in a sample where silk dominates. Instead, the β-sheet content becomes more critical in determining conductivity trends. 

When cellulose dominates in the film, this is not the case, and instead, cellulose semicrystallinity may play an important part as well as mechanical properties. It is essential to note the 75% silk samples follow the β-sheet content trend, but the 25% silk samples are flipped, meaning the 1% ethanol film with only 13.97% β-sheets has a higher ionic conductivity than the film coagulated with 10% ethanol and 17.12% β-sheets. When silk is the dominant component in the mixture, better ionic conductivity is seen compared to samples where cellulose dominates. It is also interesting to note that 25% silk films are more semicrystalline, according to the X-ray scattering profiles in Figure 6. Another possibility is due to an increase in spacing (from 0.71 to 0.75 nm) between the silk and cellulose carbon chains as a function of silk content. This increase in spacing can cause an increase in segmental motion resulting in higher ionic conductivity. For this reason, one could assume this becomes a more critical aspect of morphology in determining conductivity than β-sheet content, perhaps. Additionally, as discussed in the previous section, the elastic modulus may be an important property that drives the conductivity trend. When looking at the 25% silk samples, the sample coagulated with 10% ethanol has a higher elastic modulus, but a lower conductivity compared to the 1% ethanol sample. This may be due to the fact that since silk does not dominate, and rather cellulose does, the β-sheet content is no longer the only driving factor in conductivity. Instead, the mechanical properties are more critical. The film, which has a higher elastic modulus, more rigidity, and less flexibility, has lower ionic conductivity compared to the opposite. This makes sense as it would be more difficult for ions to move throughout a film where there is less flexibility. It is interesting to see how the biocomposite composition changes other sample characteristics that ultimately impact ionic conductivity. The findings suggest that when more silk is present, β-sheet content is the more important characteristic, but when more cellulose is present, β-sheet content does not dictate, and rather mechanical properties do.

**Figure 9:**
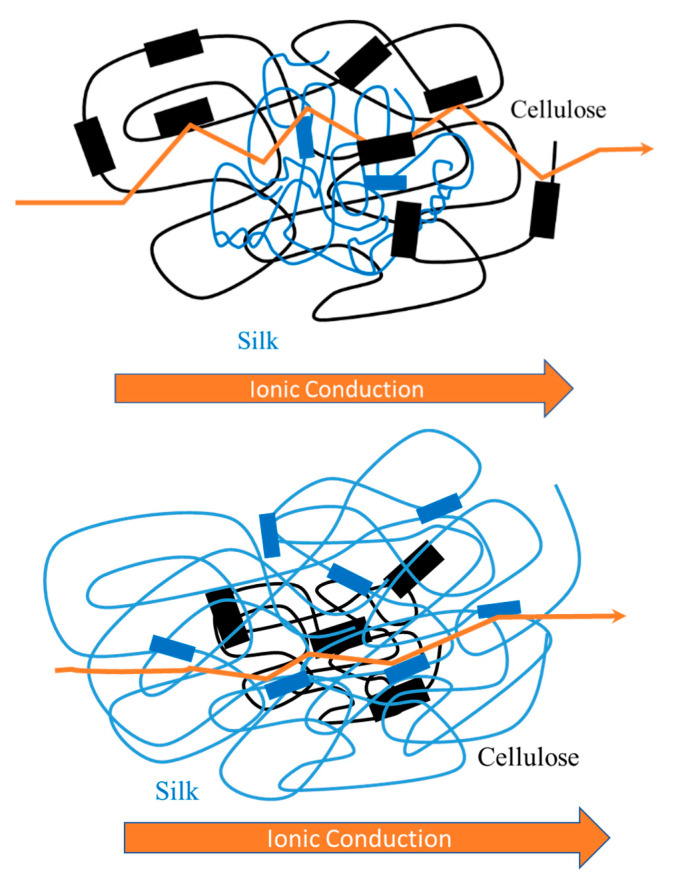
Schematic representation of ion diffusion in a solid electrolyte based on two different compositions of silk/cellulose biocomposites. The top diagram represents 25% silk, and the bottom diagram represents 75% silk.

Because the glass transition temperatures are very similar for all samples in this study, the use of a *T*_g_-normalized graph is not necessary since it would show nearly the same pattern as Figure 8. According to Ye et al., this *T*_g_-normalized graph is important in determining the effect morphology has on the system, if any [42]. If all data points from each sample collapse onto one line, this would mean the only contributing factor in the system for ionic conductivity was the glass transition temperature. However, since this is not observed in Figure 8, it can be assumed other contributing factors are affecting the ionic conductivity [42]. These factors can include morphology, which would affect the segmental motion of the polymer chains or ion hopping within the system [26,40]. As discussed previously, the data suggests samples with higher silk content follow the β-sheet content trend formerly addressed in two other papers, including a recent publication by Pereira et al. [26], which suggested β-sheets enhanced ion mobility. Additionally, the samples containing a higher content of cellulose exhibited higher cellulose semicrystallinity and followed the same trend as seen in the elastic modulus of the samples. Similarly, it can be observed for the 100% regenerated samples. This data combined suggests there is a strong correlation between the ionic conductivity and morphology of the systems, as illustrated in Figure 9. This schematic helps to illustrate why there is a difference in conductivity. When there is a higher silk content, resulting in less semicrystallinity of the polysaccharide, the ions can take a more direct path with fewer steps, resulting in ions moving more efficiently and faster through the solid biocomposite, which also results in higher conductivity. However, the opposite is true when there is lower silk content. In this biocomposite, there are more semicrystalline regions from the polysaccharide, and as a result, the ions cannot take a direct path like in the 75% silk films. This results in a slower movement of ions and a lower conductivity.

## 3. Experimental Section

### 3.1. Materials

#### 3.1.1. Ionic Liquid

The ionic liquid, 1-Ethyl-3-methylimidazolium acetate (95%), was purchased from Sigma-Aldrich. Before use, the liquid was pretreated in a vacuum oven (30 inHg) at 50 °C for 24 h. This removes any water in the ionic liquid.

#### 3.1.2. Cellulose

Avicel microcrystalline cellulose of 250 µm (Techware: Z26578-0) was purchased from Analtech and used to mix with *Bombyx mori* silk. Like the ionic liquid, the cellulose was placed in a vacuum oven (30 inHg) at 50 °C for 24 h to remove any residual water before mixing.

#### 3.1.3. Silk

Treenway Silks (Lakewood, CO, USA) was used to acquire the *Bombyx mori* silk cocoons. A 0.02 M NaHCO_3_ (Sigma-Aldrich, Saint Louis, MO, USA) solution was used to boil the silkworm cocoons for 15 min. This removed the sericin coating on the fibers, followed by rinsing with deionized water three times to ensure all sericin was adequately removed. These degummed fibers air-dried overnight. Following this, they were put into a vacuum oven (30 inHg) at room temperature to remove moisture on the surface of the fibers. 

#### 3.1.4. Dissolution of the Protein and Polysaccharide

The ionic liquid and protein/polysaccharide were measured to be specific percentages of the total mass of the biocomposite film. The ionic liquid accounted for 90% of the mass, while the protein (silk) and polysaccharide (cellulose) together were measured to be 10% by mass of the film. The solids were broken down further into individual ratios of silk and cellulose. Specifically, one set of biocomposites contained 25% silk and 75% cellulose while the other contained 75% silk and 25% cellulose, in addition to the 100% silk and 100% cellulose samples. The pretreated ionic liquid was placed in a vial and then placed into a silica oil bath held at 80 °C. The dissolution process began by first adding silk to the vial, followed by cellulose. When both materials were fully dissolved in the ionic liquid, the solution was left to mix for 24 h at 80 °C. 

#### 3.1.5. Preparation of Regenerated Biofilm

After 24 h of continuous mixing, the gel solution was transferred into 12 mm × 12 mm × 1 mm polylactic acid 3-D printed molds. Specifically, 1 mL micropipette tips were first heated to 75 °C to ensure the solution did not solidify inside the tip. Then, the solution was pipetted into the molds. Once filled, each mold was placed in 100 mL of coagulation agent inside a 250 mL beaker and sealed with parafilm for 48 h. The ethanol used in this experiment was purchased from Fisher Scientific (Waltham, MA, USA). This step removes as much ionic liquid as possible from the film as well as regenerates the natural polymers. Once 48 h had passed, each mold was removed and rinsed three times with distilled water to remove as much residual ionic liquid on the surface as possible. The molds were then transferred to Teflon Petri dishes and allowed to dry in a low-pressure desiccator.

### 3.2. Characterization

#### 3.2.1. Fourier Transform Infrared Spectroscopy

Fourier Transform Infrared Spectroscopy (FTIR) analysis was performed using a Bruker (Billerica, MA, USA) ALPHA-Platinum ATR-FTIR Spectrometer with Platinum-Diamond sample module. Before any background scans and between each sample, acetone was used to clean the FTIR diamond and hammer. Then, 32 sample scans in 6 different locations of the biocomposite were performed after 128 background scans. Following this, the amide I region (1595 cm^−1^–1705 cm^−1^) was studied using Fourier self-deconvolution. Specifically, Lorentzian line shape with a noise reduction factor of 0.3 and 25.614 cm^−1^ half-bandwidth was utilized for deconvolution. Fitting the results and integrating to find the area correlation to a particular wavelength was performed using Gaussian profiles. These analyses were run using Opus 7.2 software. After Fourier self-deconvolution, min-max normalization was used to normalize the data from 4000 cm^−1^ to 400 cm^−1^ to highlight the functional groups better.

#### 3.2.2. Scanning Electron Microscopy

A JEOL (Akishima, Tokyo, Japan) JCM-6000 SEM was used for Scanning Electron Microscopy (SEM). The topography of the biocomposites was studied in the resulting images. A magnification of 200× was used to acquire images. Au-Pt coating was deposited onto the films to lessen the buildup of surface charge during imaging. The conductive coating was applied using a DII-29010SCTR Smart Coater at vacuum level 4 Pa with a deposition time of 60 s.

#### 3.2.3. Thermogravimetric Analysis

TA Instruments (New Castle, DE, USA) Discovery TGA system was utilized to perform Thermogravimetric analysis (TGA) with 5 mg samples under a nitrogen gas purge of 25 mL/minute. The run was started at 30 **°**C, followed by an isothermal period of one minute, and ramped 10 °C per minute to 600 °C. The furnace was allowed to cool to 30 °C in between each sample run. The data was analyzed using step transition analyses and derivative plots. Using these analysis techniques allowed the weight-loss percentage, temperature corresponding to the decomposition of the sample at its highest rate (*T*_∆Max_), as well as the onset temperature of decomposition (*T*_Onset_), to be determined.

#### 3.2.4. Differential Scanning Calorimetry

5 mg samples encapsulated in aluminum Tzero pans were analyzed under a nitrogen gas flow of 50 mL per minute using the TA Instruments Differential Scanning Calorimetry (DSC). This was equipped with a refrigerated cooling system. Before running any samples, indium was used for DSC calibration for temperature and heat flow, and calibration of the heat capacity and heat flow was performed using sapphire and aluminum references. The samples were first equilibrated to room temperature, isothermal for 10 min, ramped 10.00 °C per minute to 120 °C, isothermal for 10 min to remove bound solvents, ramped 10.00 °C per minute back to −30 °C, isothermal for another 10 min, and ramped 10.00 °C per minute to 275 °C. 

#### 3.2.5. X-Ray Scattering

Dual Source and Environmental X-ray Scattering (DEXS) Xeuss 2.0 from Xenocs (Holyoke, MA, USA) was used to perform X-ray scattering under vacuum and at room temperature. Prior to running the samples, they were placed in a desiccator and then cut into squares that cover the sample holder. A high flux collimation with a 1.2 mm × 1.2 mm slot was used with a 600 s run time for the 75% silk and 100% silk samples, while 300 s was used for the 25% silk and 100% cellulose samples. Foxtrot 3.4.9 was used to evaluate the X-ray scattering profiles, and ultimately, azimuthal integration was used on the isotropic 2-D scattering patterns to yield intensity versus scattering vector.

#### 3.2.6. Atomic Force Microscopy (Nanoindentation)

The elastic modulus of four silk-cellulose composites was measured at 120 °C via Atomic Force Microscopy (AFM). The samples were adhered to steel disks using silver paste and left in a fume hood to dry and to prevent adhesive vapor from depositing onto the sample surface. Subsequently, the samples were stored in a vacuum desiccator to keep the samples in a dehydrated state. An Asylum Research (Goleta, CA, USA) MFP-3D equipped with a Nanotools Biosphere BFP-40 AFM was employed for both topographical scans and nanoindentation measurements. The inverse optical lever sensitivity (InVols) of the AFM probe was determined by performing nanoindentation on a clean disk of mica and measuring the slope of the deflection versus cantilever position curve. The spring constant of the probe was extrapolated from its thermal oscillation spectrum. Prior to measurement, the samples were magnetically mounted onto a heating plate integrated into the AFM. The sample was allowed to heat at a rate of 5 °C per minute up to 120 °C. Subsequently, a 20 µm × 20 µm topographical scan was performed, followed by nanoindentation measurements at 25 different locations. The topographical scan was done in tapping mode to prevent deformation by the tip. A force versus load-displacement curve was obtained from each nanoindentation, and the elastic moduli were extracted using the Johnson-Kendall-Roberts (JKR) model. The JKR model is a modification of the Hertzian model that accounts for the adhesion between the sample and tip during nanoindentation [43]. The JKR model is typically used when measuring soft materials where adhesion can strongly influence the determination of elastic properties [44]. While both the loading and unloading force versus indentation curves were collected, only the loading curve was considered during analysis. The JKR model is considered most accurate when applied to the loading curve when using AFM based systems [45]. The analysis tool built into the Asylum MFP-3D software package (v. 31) was utilized for all regression analyses. 

#### 3.2.7. Dielectric Relaxation Spectroscopy

Dielectric Relaxation Spectroscopy (DRS) was used to determine the ionic conductivity of the biocomposites made with different compositions and coagulation agents and was performed at the University of Pennsylvania. The film was placed between two stainless steel electrodes, the top plate having a diameter of 6 mm, and then placed in a Janis (Woburn, MA, USA) VPF-100 cryostat under vacuum [46]. Solartron Modulab XM materials test system was utilized in a temperature range of 300 K–450 K (26.85–176.85 °C), with the measurements starting at 450 K to ensure no excess water remained in the biocomposite. Additionally, the samples were tested over a frequency range of 0.1 Hz to 1 MHz [46]. After each measurement, the temperature was decreased by 10 K.

## 4. Conclusions

Systematic variation of silk/cellulose ratios and coagulation agents provided evidence to suggest a direct relationship between ionic conductivity and morphology. Specifically, the morphological, thermal, mechanical, and ionic conductivity properties of the samples were altered. Secondary structure differences are seen as results of FTIR, with the two samples coagulated with 10% ethanol having the highest β-sheet content, followed by the 1% ethanol samples. When investigating thermal properties, it was interesting to see that when the composition varied, the thermogram showed a tendency to have a comparable number of peaks to the dominating biopolymer. Films where cellulose dominated showed a trimodal thermogram, similar to the 100% regenerated cellulose film, and films with more silk showed a bimodal thermogram, which was one peak more than the single peaked thermogram seen in the 100% regenerated silk sample. This illustrates the influence of composition on the system. In addition, X-ray scattering data showed samples with only 25% silk and 75% cellulose were more semicrystalline than those with a higher percentage of silk. This suggests the semicrystallinity of the system is affected by silk versus cellulose content. The results from X-ray scattering, AFM based nanoindentation, and FTIR correlate with the ionic conductivity of the silk/cellulose biocomposite films. When there is higher silk content, there are fewer semicrystalline regions from the polysaccharide, and therefore ions can move more directly through the structure leading to higher conductivity. In this system, ionic conductivity is related to β-sheet content. The higher the β-sheet content, the higher the ionic conductivity. The opposite is true, where a higher cellulose content leads to more semicrystalline regions, not allowing ions to move as efficiently through the biocomposite. This leads to lower conductivity. Future studies will need to be completed to investigate this trend further, but this work demonstrates that ionic conductivity can be tuned for specific needs in biocomposite films, which may be necessary for modern materials science as well as medicine.

## Figures and Tables

**Table 1:** Secondary structure contents of the 100% regenerated silk sample, and 25% silk and 75% silk biocomposites.

Composition	Coagulation	Side Chains	β-Sheets	Random Coils	Alpha Helices	Turns
75/25 Silk-Cellulose	10% EtOH	0.92%	20.56%	29.16%	18.37%	30.98%
75/25 Silk-Cellulose	1% EtOH	0.44%	18.24%	27.14%	22.40%	31.77%
25/75 Silk-Cellulose	10% EtOH	1.08%	17.12%	33.36%	19.42%	29.02%
25/75 Silk-Cellulose	1% EtOH	1.04%	13.97%	38.83%	17.27%	28.89%
100 Silk	100% EtOH	5.71%	46.71%	15.36%	10.93%	21.29%

**Table 2:** Start and end temperatures, total weight-loss percentage, and the maximum temperature of the derivative are used to characterize thermal gravimetric analysis (TGA) results of the 100% regenerated samples and varied composition samples.

Composition	Coagulation	*T*_Onset_ (°C)	*T*_End_ (°C)	*Wt. Loss* (%)	*T*_∆Max_ (°C)
75/25 Silk-Cellulose	10% EtOH	225.8	319.6	67.95	229.5, 285.8
75/25 Silk-Cellulose	1% EtOH	213.8	310.2	67.63	233.4, 285.0
25/75 Silk-Cellulose	10% EtOH	223.4	305.6	65.95	243.3, 281.2, 317.0
25/75 Silk-Cellulose	1% EtOH	219.2	314.5	67.86	240.9, 280.7, 316.9
100 Silk	100% EtOH	251.5	305.4	53.48	278.1
100 Cellulose	100% H_2_O	219.9	295.0	64.74	238.2, 285.3, 317.5

**Table 3:** Glass transition temperatures of varied composition films and 100% regenerated samples, determined by DSC in degrees Celsius and Kelvin.

*Composition*	*Coagulation*	*T*_g_ (°C)	*T*_g_ (K)
75/25 Silk-Cellulose	10% EtOH	128.58	401.73
75/25 Silk-Cellulose	1% EtOH	129.10	402.25
25/75 Silk-Cellulose	10% EtOH	133.07	406.22
25/75 Silk-Cellulose	1% EtOH	127.30	400.45
100 Silk	100% EtOH	137.98	411.13
100 Cellulose	100% H_2_O	128.68	401.83

**Table 4:** Scattering vector and correlation distances for two varied composition silk/cellulose biocomposites coagulated with two different coagulation agents.

Peak Position	q (nm^−1^)			
	d (nm)			
	25/75 Silk-Cellulose		75/25 Silk-Cellulose	
	1% EtOH	10% EtOH	1% EtOH	10% EtOH
1	8.83 0.71	8.83 0.71	8.40 0.75	8.40 0.75
2	14.40 0.44	14.40 0.44	14.40 0.44	14.40 0.44
3	20.27 0.31	20.27 0.31	20.27 0.31	20.27 0.31
4	25.15 0.25	25.15 0.25	24.79 0.23	24.79 0.23
5	29.43 0.21	29.43 0.21	29.43 0.21	29.43 0.21

**Table 5:** Scattering vector and correlation distances for regenerated 100% cellulose and 100% silk samples coagulated with water and ethanol, respectively.

Peak Position	q (nm−1) d (nm)	Sample	Peak Position	q (nm−1) d (nm)	Sample
a	5.42 1.16	Regenerated 100% Silk	g	1.31 4.80	Regenerated 100% Cellulose
b	14.45 0.44		h	8.78 0.72	
c	17.67 0.36		i	14.32 0.44	
d	22.52 0.28		j	15.66 0.40	
e	28.66 0.22		k	20.56 0.30	
f	31.40 0.20		l	24.9 0.25	
			m	29.66 0.21	

## References

[1] Pavlovic M. (2015). What Are Biomaterials?. Bioengineering: A Conceptual Approach.

[2] Jia X., Wang C., Zhao C., Ge Y., Wallace G.G. (2016). Toward biodegradable Mg–air bioelectric batteries composed of silk fibroin–polypyrrole film. Adv. Funct. Mater..

[3] Agarwal V., Huber G.W., Conner W.C., Auerbach S.M. (2011). Simulating infrared spectra and hydrogen bonding in cellulose Iβ at elevated temperatures. J. Chem. Phys..

[4] Wilson D.B., Irwin D.C. (1999). Genetics and properties of cellulases. Recent Progress in Bioconversion of Lignocellulosics.

[5] Yamashiki T., Matsui T., Saitoh M., Matsuda Y., Okajima K., Kamide K., Sawada T. (1990). Characterisation of cellulose treated by the steam explosion method. Part 3: Effect of crystal forms (cellulose I, II and III) of original cellulose on changes in morphology, degree of polymerisaion, solubility and supermolecular structure by steam explosion. Br. Polym. J..

[6] Helbert W., Nishiyama Y., Okano T., Sugiyama J. (1998). Molecular imaging ofhalocynthia papillosacellulose. J. Struct. Biol..

[7] Cheng G., Varanasi P., Li C., Liu H., Melnichenko Y.B., Simmons B.A., Kent M.S., Singh S. (2011). Transition of cellulose crystalline structure and surface morphology of biomass as a function of ionic liquid pretreatment and its relation to enzymatic hydrolysis. Biomacromolecules.

[8] Dumitriu S. (2004). Polysaccharides: Structural Diversity and Functional Versatility.

[9] Hu X., Cebe P., Weiss A.S., Omenetto F., Kaplan D.L. (2012). Protein-based composite materials. Mater. Today.

[10] Zhou L., Wang Q., Wen J., Chen X., Shao Z. (2013). Preparation and characterization of transparent silk fibroin/cellulose blend films. Polymer.

[11] Marsh R.E., Corey R.B., Pauling L. (1955). An investigation of the structure of silk fibroin. Biochim. Biophys. Acta.

[12] Takahashi Y., Gehoh M., Yuzuriha K. (1999). Structure refinement and diffuse streak scattering of silk (Bombyx mori). Int. J. Biol. Macromol..

[13] Chen X., Knight D.P., Shao Z., Vollrath F. (2001). Regenerated Bombyx silk solutions studied with rheometry and FTIR. Polymer.

[14] Chen X., Shao Z., Marinkovic N.S., Miller L.M., Zhou P., Chance M.R. (2001). Conformation transition kinetics of regenerated Bombyx mori silk fibroin membrane monitored by time-resolved FTIR spectroscopy. Biophys. Chem..

[15] Stanton J., Xue Y., Pandher P., Malek L., Brown T., Hu X., Salas-de la Cruz D. (2018). Impact of ionic liquid type on the structure, morphology and properties of silk-cellulose biocomposite materials. Int. J. Biol. Macromol..

[16] Stanton J., Xue Y., Waters J.C., Lewis A., Cowan D., Hu X., Salas-de la Cruz D. (2017). Structure–property relationships of blended polysaccharide and protein biomaterials in ionic liquid. Cellulose.

[17] Hadadi A., Whittaker J.W., Verrill D.E., Hu X., Larini L., Salas-De La Cruz D. (2018). A Hierarchical Model to Understand the Processing of Polysaccharides/Protein-Based Films in Ionic Liquids. Biomacromolecules.

[18] Blessing B., Trout C., Morales A., Rybacki K., Love S.A., Lamoureux G., O’Malley S.M., Hu X., Salas-de la Cruz D. (2019). Morphology and ionic conductivity relationship in silk/cellulose biocomposites. Polym. Int..

[19] Zhang H., Wu J., Zhang J., He J. (2005). 1-Allyl-3-methylimidazolium chloride room temperature ionic liquid: A new and powerful nonderivatizing solvent for cellulose. Macromolecules.

[20] Johnson K.E. (2007). What’s an ionic liquid?. Interface Electrochem. Soc..

[21] Pinkert A., Marsh K.N., Pang S., Staiger M.P. (2009). Ionic liquids and their interaction with cellulose. Chem. Rev..

[22] Freddi G., Romanò M., Massafra M.R., Tsukada M. (1995). Silk fibroin/cellulose blend films: Preparation, structure, and physical properties. J. Appl. Polym. Sci..

[23] Love S.A., Popov E., Rybacki K., Hu X., Salas-de la Cruz D. (2020). Facile treatment to fine-tune cellulose crystals in cellulose-silk biocomposites through hydrogen peroxide. Int. J. Biol. Macromol..

[24] Salas-de la Cruz D. (2011). Morphology and Ionic Conductivity of Polymerized Ionic Liquids.

[25] Murphy E. (1960). The dependence of the conductivity of cellulose, silk and wool on their water content. J. Phys. Chem. Solids.

[26] Pereira R.F., Brito-Pereira R., Gonçalves R., Silva M.P., Costa C.M., Silva M.M., de Zea Bermudez V.n., Lanceros-Méndez S. (2018). Silk fibroin separators: A step toward lithium-ion batteries with enhanced sustainability. ACS Appl. Mater. Interfaces.

[27] Hu X., Kaplan D., Cebe P. (2006). Determining beta-sheet crystallinity in fibrous proteins by thermal analysis and infrared spectroscopy. Macromolecules.

[28] Um I.C., Kweon H., Park Y.H., Hudson S. (2001). Structural characteristics and properties of the regenerated silk fibroin prepared from formic acid. Int. J. Biol. Macromol..

[29] He S.-J., Valluzzi R., Gido S.P. (1999). Silk I structure in Bombyx mori silk foams. Int. J. Biol. Macromol..

[30] Gong Z., Huang L., Yang Y., Chen X., Shao Z. (2009). Two distinct [small beta]-sheet fibrils from silk protein. Chem. Commun..

[31] Asakura T., Yamane T., Nakazawa Y., Kameda T., Ando K. (2001). Structure of Bombyx mori silk fibroin before spinning in solid state studied with wide angle x-ray scattering and 13C cross-polarization/magic angle spinning NMR. Biopolymers.

[32] Asakura T., Okushita K., Williamson M.P. (2015). Analysis of the Structure of Bombyx mori Silk Fibroin by NMR. Macromolecules.

[33] Liu X., Zhang K.-Q. (2014). Silk Fiber—Molecular Formation Mechanism, Structure-Property Relationship and Advanced Applications.

[34] Saitoh H., Ohshima K.-i., Tsubouchi K., Takasu Y., Yamada H. (2004). X-ray structural study of noncrystalline regenerated Bombyx mori silk fibroin. Int. J. Biol. Macromol..

[35] Nieduszynski I., Preston R. (1970). Crystallite size in natural cellulose. Nature.

[36] Eyley S., Thielemans W. (2014). Surface modification of cellulose nanocrystals. Nanoscale.

[37] French A.D. (2014). Idealized powder diffraction patterns for cellulose polymorphs. Cellulose.

[38] Fernandes A.N., Thomas L.H., Altaner C.M., Callow P., Forsyth V.T., Apperley D.C., Kennedy C.J., Jarvis M.C. (2011). Nanostructure of cellulose microfibrils in spruce wood. Proc. Natl. Acad. Sci. USA.

[39] Kafle K., Shin H., Lee C.M., Park S., Kim S.H. (2015). Progressive structural changes of Avicel, bleached softwood, and bacterial cellulose during enzymatic hydrolysis. Sci. Rep..

[40] Salas-de la Cruz D., Green M.D., Ye Y., Elabd Y.A., Long T.E., Winey K.I. (2012). Correlating backbone-to-backbone distance to ionic conductivity in amorphous polymerized ionic liquids. J. Polym. Sci. Part B Polym. Phys..

[41] Lee M., Choi U.H., Salas-de la Cruz D., Mittal A., Winey K.I., Colby R.H., Gibson H.W. (2011). Imidazolium polyesters: Structure–property relationships in thermal behavior, ionic conductivity, and morphology. Adv. Funct. Mater..

[42] Ye Y., Elabd Y.A. (2011). Anion exchanged polymerized ionic liquids: High free volume single ion conductors. Polymer.

[43] Johnson K.L., Kendall K., Roberts A. (1971). Surface energy and the contact of elastic solids. Proc. R. Soc. Lond. A Math. Phys. Sci..

[44] Ebenstein D.M., Pruitt L.A. (2006). Nanoindentation of biological materials. Nano Today.

[45] Notbohm J., Poon B., Ravichandran G. (2012). Analysis of nanoindentation of soft materials with an atomic force microscope. J. Mater. Res..

[46] Griffin P.J., Freyer J.L., Han N., Geller N., Yin X., Gheewala C.D., Lambert T.H., Campos L.M., Winey K.I. (2018). Ion Transport in Cyclopropenium-Based Polymerized Ionic Liquids. Macromolecules.

